# Depression and Insomnia of Front-Line Medical Staff During the COVID-19 Outbreak in China: An On-Line Cross-Sectional Study

**DOI:** 10.3389/fpsyg.2022.897896

**Published:** 2022-06-29

**Authors:** Donglin Zhang, Hailong Luo, Litian Xiao, Zhun Zhang, Jianqing Huang, Xiaoqin Li, Hongke Zhu, Cuiwei Lai

**Affiliations:** ^1^Department of Emergency Medicine, Meizhou People's Hospital, Meizhou, China; ^2^Department of Rehabilitation Medicine, Meizhou People's Hospital, Meizhou, China; ^3^College of Education, Guangzhou University, Guangzhou, China; ^4^Nursing Department, Meizhou People's Hospital, Meizhou, China

**Keywords:** COVID-19, medical staff, depression, insomnia, influencing factors

## Abstract

**Purpose:**

During the COVID-19 outbreak, medical staff working in high-risk workplaces had a higher rate of epidemic infection. They also faced heavy workloads and pressure, which means they are more likely to suffer from psychological problems than others. To understand the mental health of medical staff during the epidemic, we explore the characteristics of medical staff susceptible to negative psychological emotions during the outbreak of public safety and health events. At the same time, we provide corresponding prevention and intervention measures to help them relieve negative emotions, this study compared the psychological symptoms of front-line and non-front-line medical staff, then explored its influencing factors.

**Methods:**

This research investigated 5,924 medical staff in Guangdong, Beijing, Hubei, Hainan, Jiangxi, and Henan in China. The questionnaires were released online by Questionnaire Star, and levels of depression, anxiety, insomnia, and social support were measured by Patients' Health Questionnaire Depression Scale-9 item (PHQ-9), Generalized Anxiety Disorder 7-item Scale (GAD-7), Insomnia Severity Index (ISI) and Social Support Rating Scale (SSRS).

**Results:**

The depressive, anxious, and insomniac scores of front-line medical staff during the COVID-19 epidemic period were higher than those of non-front-line with significant differences (*P* < 0.001). In addition, front-line nurses went through the most serious psychological problems. Age, education, and anxiety level were the influencing factors of depression and insomnia in front-line medical staff. Among them, age was a protective factor for depression (OR = 0.71 <1, *P* = 0.001 <0.05, Beta = −0.34), while educational level was a risk factor for insomnia (OR = 1.27, *P* = 0.02 <0.05, Beta = 0.24).

**Conclusions:**

During the epidemic, front-line medical staff would experience more serious psychological problems, especially those who were younger, less experienced, and insufficiently educated. Attention should be paid to giving them psychological assistance and psychological interventions in the future.

## Introduction

In 2020, COVID-19 rapidly spread around the world. The epidemic has attracted the attention of the international community as a public health emergency. Since the outbreak, individuals and governments have responded to this major public health emergency, bringing in measures that have affected the lives of hundreds of millions of people, changing how people socialize, work, study, and live (Wu et al., 2021).

According to a UN report, COVID-19 not only harms physical health but also increases psychological distress (United Nations, 2020). Some researchers have declared that the fear of being infected, increased work stress, changes in lifestyle, and deterioration of living conditions may lead to anxiety, sleep disorders, depression, and other mental problems (Pulvirenti et al., 2020; Zhang J. et al., 2020). The results of several studies investigating the influence of infectious diseases such as SARS and Ebola showed that, during major public health emergencies, medical staff and other populations might go through mental problems (Lu et al., 2006; Matsuishi et al., 2012; Wing and Leung, 2012; Jeong et al., 2016; Kisely et al., 2020). Previous studies have demonstrated that when working in high-stress and high-risk epidemic environments, medical staff face huge psychological burdens and psychological barriers (Lin et al., 2007; Lehmann et al., 2015). Those medical staff fighting on the front lines, not only have to overcome enormous pressure but face the risk and fear of being infected and worry about their colleagues and family members as well, meaning they are much more prone to mental problems (Zhang J. et al., 2020).

Relevant evidence has been provided to support the points of view outlined above. For example, a study of 2,182 Chinese patients indicated that the anxiety and insomnia levels of medical staff were significantly higher than those of non-medical staff (Zhang W. R. et al., 2020). Meanwhile, the result of a research about medical staff in Wenzhou, China, in which investigators compared the prevalence and severity of anxiety, depression, and insomnia among front line and non-front line medical staff, illustrated that the levels of anxiety, depression, and insomnia of medical staff were significantly higher than those of non-front-line, and the self-reported occupational stress of front-line medical staff was higher (Zhang J. et al., 2020).

In addition, some scholars have proposed that people with different social roles and different health conditions may be susceptible to different types and severities of mental problems (Tan et al., 2020). A recent study indicated that not all healthcare workers were affected equally by the COVID-19 outbreak and that a group of nurses suffered many more psychological problems especially (Lai et al., 2019). Several findings have verified the conclusions, which were consistent with those mentioned above, observing that nurses reported higher exhaustion/stress, depressive symptoms, and lower job fulfillment compared with physicians during the COVID-19 pandemic (Zerbini et al., 2020). It is therefore particularly important to pay attention to the possible mental problems of medical staff during the epidemic and to develop corresponding measures to prevent and intervene in susceptible populations.

Previous studies have only focused on comparing the mental health of front-line and non-front-line medical staff in one region of China. This focus does not comprehensively consider the situation in multiple regions. In addition, there is no study comparing the mental status of front-line doctors and nurses. Therefore, based on earlier studies, this research explores the mental health status of front-line and non-front-line medical staff during the epidemic in several regions of China and examines the psychological differences between doctors and nurses, as well as the factors causing such differences.

## Materials and Methods

### Participants

This study was approved by the Ethics Committee of Meizhou People's Hospital (2020-C-119). We explained the study to all participants and obtained their informed consent. Between February 18 and March 18, 2020, we used a simple sampling method to investigate medical staff in Guangdong, Beijing, Hubei, Hainan, Jiangxi, and Henan in China by releasing questionnaires to WECHAT clients in the form of “Questionnaire Star.” The online survey included measures of depression, anxiety, insomnia, social support, and a short sociodemographic questionnaire. A total of 6,044 medical staff participated in the study. The inclusion criteria were: (1) medical staff (doctors and nurses) who worked at hospitals during the epidemic period of COVID-19; (2) those willing to participate in the study with informed consent; and (3) people who completed the questionnaire independently. The exclusion criteria were participants who provided inconsistent answers to the same questions. After excluding invalid questionnaires, 5,924 samples participated in the study, including 2,546 doctors and 3,378 nurses. The effective rate of the questionnaire was 98.01%. In this study, medical staff who had worked in high-risk locations, such as fever outpatients, infectious diseases departments, emergency rooms, ICU, respiratory departments, or general practice were classified as “Front-Line”(FL). Among the 5,924 participants, 2,469 were “front-line medical staff,” and 3,455 were “non-front-line medical staff.”

### Assessment Instruments

#### Demographic Questionnaire

The basic background information of the subjects was obtained using a population survey questionnaire. It included questions such as gender, age, education level, and the hospital departments where they worked. It also asked whether the participants smoke, drink, or had any kind of physical diseases.

#### Patients' Health Questionnaire Depression Scale-9 Item (PHQ-9)

PHQ-9 (Kroenke et al., 2001) is a 9-item questionnaire used to measure depression, which is the quantitative assessment criteria recommended by the Diagnostic Statistical Manual of Mental Disorders (DSMV), fifth edition, published by the American Psychiatric Association (APA) in May 2013. It is also a Likert 4-point scale, with the scores ranging from 0 to 3 (0: not at all, 3: almost every day). The total score ranges from 0 to 27 points, 6 to 9 are mildly depressed, 10–14 are moderately depressed, 15–19 have severe depression, and 20–27 are very severe. For the convenience of further research, scores from 0 to 5 are defined as no depression, and scores above 5 are depression in this study. The scale has been verified by domestic and foreign studies with good reliability and validity (Spitzer et al., 2006; Ruiz et al., 2011).

#### Generalized Anxiety Disorder 7-Item Scale (GAD-7)

GAD-7 (Lowe et al., 2008) is utilized to assess anxiety with 7 items. Due to its diagnostic reliability, factorial, construct, and criterion validity, the GAD-7 is one of the most widely used anxiety measures, both in clinical practice and research. It can be used to screen, diagnose and assess the severity of anxiety disorders, and measure social phobia, post-traumatic stress disorder, and panic disorder as well (Toussaint et al., 2020). A Likert 4-point scale was used, and the total score ranged from 0 to 21 points, which is normal if the scores are <5, mild anxiety if it was 6 to 9, moderate anxiety between 10 to 14, and severe anxiety if it was more than 15.

#### Insomnia Severity Index (ISI)

ISI is composed of 7 items, which mainly measure the sleep quality of the subjects. ISI was established by Morin et al. with good reliability and validity (Bastien et al., 2001). The scoring method of this scale is similar to that of GAD-7, with the higher scores indicating more serious insomnia. Scores of 5 to 8 points are mild insomnia, 9 to 15 points are moderate, and above 16 points are severe.

#### Social Support Rating Scale (SSRS)

The SSRS (Xiao, 1994) established by Xiao Shuiyuan in 1994 is used to evaluate the social support of medical staff in this research because of the specific environmental and cultural conditions in China. It contains 10 items that measure three dimensions of social support, including 4 items of subjective support, 3 items of objective support, and 3 items of utilization of support. The total score ranges from 12 to 66 points with a subjective support score ranging from 8 to 32, an objective support score ranging from 1 to 22, and a utilization of support score ranging from 3 to 12. Higher scores mean higher social support. This scale has been applied by a large number of Chinese researchers due to its high reliability and validity (Gao et al., 2009; Xie et al., 2009).

### Statistical Analysis Methods

SPSS 24.0 was used for statistical analysis. Firstly, a descriptive statistical analysis was conducted on the demographic information and scores of each scale of front-line and non-front-line medical staff respectively, and then the discrepancy tests were conducted. The categorical variables were analyzed using a Kruskal-Wallis H test for the categorical variables. After that, A general linear model was used for continuous variables, age, sex, and education as covariates. Finally, Binary logistic regression was conducted to explore the influencing factors of problems of front-line medical staff. Adjusted odds ratios (ORs) with 95% confidence intervals (CIs) were provided. The level of significance was set as 2-tailed P values of 0.05.

## Results

### Common Method Deviation Test

In this study, a self-reported questionnaire was used for measurement, meaning there may be a common method deviation. Harman's single-factor test (Podsakoff et al., 2003) was used to measure whether there was a serious common method bias effect. Factor analysis was conducted on all items, and a total of 14 common factors with characteristic roots >1 were extracted. The first common factor explained the total variance of 31.89%, which was far less than the 40% critical standard (Zhou and Long, 2004), indicating that there was no serious common method deviation problem.

### Demographic Information of All Participants

Most of the subjects were female, with a total male to female ratio of 1:2.6 (1645:4279), among which the male to female ratio of front-line medical staff was 1:1.96 (833:1636), and that of non-front-line medical staff was 1:3.25 (812:2643). Demographic information on the age and education level of the medical staff is shown in Table 1. The age group options 1–6 on the demographic questionnaire represent ages under 20, 21–30, 31–40, 41–50, 51–60, and over 60 respectively. Items 1 to 6 represent educational level mean below high schools, technical secondary schools, junior colleges, undergraduates, masters, and doctoral degrees separately. As shown in Table 1, the age of medical staff was concentrated in 20–40 years old, and most of them were over 30 years old. A large proportion of them graduated from junior colleges or universities.

**Table 1:** Demographic information of front-line and non-front-line medical staff.

**Variables**		**FL**		**NL**	
		**First-line doctors (%)**	**First-line nurses (%)**	**Non-first-line doctors (%)**	**Non-first-line nurses (%)**
Sex	Male	786 (13.3)	47 (0.8)	772 (13.0)	40 (0.7)
	Female	264 (4.5)	1,372 (23.2)	724 (12.2)	1,919 (32.4)
Age	1: ≤ 20	2 (0.0)	47 (0.8)	3 (0.1)	59 (1.0)
	2:21~30	249 (4.2)	859 (14.5)	382 (6.4)	1,030 (17.4)
	3:31~40	392 (6.6)	293 (4.9)	443 (7.5)	461 (7.8)
	4:41~50	344 (5.8)	202 (3.4)	522 (8.8)	384 (6.5)
	5:51~60	60 (1.0)	17 (0.3)	132 (2.2)	23 (0.4)
	6:>60	3 (0.1)	1 (0.0)	14 (0.2)	2 (0.0)
Education	1:below high schools	5 (0.1)	2 (0.0)	15 (0.3)	9 (0.2)
	2:Technical secondary school	121 (2.0)	399 (6.7)	228 (3.8)	612 (10.3)
	3:junior colleges	384 (6.5)	761 (12.8)	599 (10.1)	1,022 (17.3)
	4:Undergraduate	458 (7.7)	257 (4.3)	559 (9.4)	315 (5.3)
	5:Masters	73 (1.2)	0 (0.0)	81 (1.4)	1 (0.0)
	6:doctoral degrees	9 (0.2)	0 (0.0)	14 (0.2)	0 (0.0)
Smoking	Smoking.	207 (3.5)	28 (0.5)	213 (3.6)	19 (0.3)
	Don't smoke	843 (14.2)	1,391 (23.5)	1,283 (23.5)	1,940 (35.6)
Drink	Yes	100 (1.7)	22 (0.4)	110 (1.9)	20 (0.3)
	No	950 (16)	1,397 (23.6)	1,386 (23.4)	1,939 (32.7)

*FL, front-line medical staff; NL, non-front-line medical staff*.

### Comparison and Partial Correlation of Various Scale Scores of Front-Line and Non-Front-Line Medical Staff

Independent sample *t*-test was performed on 2,469 front-line medical staff samples and 3,455 non-front-line medical staff samples, as shown in Table 2, in the PHQ-9 (depression level), GAD-7 (anxiety level), and ISI (insomnia status) scales. In terms of scores, there was a significant difference between the two (*p* < 0.001), but in the score of the SSRS (social support) scale, there was no significant difference between the two (*p* = 0.71>0.05). The PHQ-9, GAD-7, and ISI scale scores of front-line medical staff were significantly higher than those of non-front-line medical staff. After controlling the effects of sex, age, education, smoking, and drinking, GAD-7, PHQ-9, ISI, and SSRS were significantly correlated (*p* < 0.001), as shown in Table 3 (first-line) and Table 4 (non-first-line).

**Table 2:** The scores of each scale of FL and NL and their comparison.

	**FL**	**NL**	**z/t**	***P***
	**(*n =* 2,469)**	**(*n =* 3,455)**		
PHQ-9	4.70 ± 4.91	3.98 ± 4.49	28.24	<0.001
GAD-7	4.29 ± 4.55	3.79 ± 4.26	20.68	<0.001
ISI	6.99 ± 6.09	5.82 ± 5.45	59.79	<0.001
SSRS	41.07 ± 9.19	41.35 ± 9.01	0.138	0.71

*NL, non-front-line medical staff. FL, front-line medical staff*.

**Table 3:** The partial correlation of each scale of first-line doctors and nurses.

	**GAD-7_**D**_**	**PHQ-9 _**D**_**	**ISI _**D**_**	**SSRS _**D**_**		**GAD-7 _**N**_**	**PHQ-9 _**N**_**	**ISI _**N**_**	**SSRS _**N**_**
GAD-7 _D_	1.00				GAD-7 _N_	1.00			
PHQ-9 _D_	0.79^***^	1.00			PHQ-9 _N_	0.79^***^	1.00		
ISI _D_	0.69^***^	0.74^***^	1.00		ISI _N_	0.69^***^	0.76^***^	1.00	
SSRS _D_	−0.28^***^	−0.39^***^	−0.33^***^	1.00	SSRS _N_	−0.29^***^	−0.39^***^	−0.33^**^	1.00

** *p < 0.01*

*** *p < 0.001; D, first-line doctors; N, first-line nurses*.

**Table 4:** The partial correlation of each scale of non-first-line doctors and nurses.

	**GAD-7_**D**_**	**PHQ-9 _**D**_**	**ISI _**D**_**	**SSRS _**D**_**		**GAD-7 _**N**_**	**PHQ-9 _**N**_**	**ISI _**N**_**	**SSRS _**N**_**
GAD-7 _D_	1.00				GAD-7 _N_	1.00			
PHQ-9 _D_	0.78^***^	1.00			PHQ-9 _N_	0.78^***^	1.00		
ISI _D_	0.67^***^	0.73^***^	1.00		ISI _N_	0.67^***^	0.76^***^	1.00	
SSRS _D_	−0.27^***^	−0.35^***^	−0.29^***^	1.00	SSRS _N_	−0.29^***^	−0.39^***^	−0.32^***^	1.00

*** *p < 0.001; D, non-first-line doctors; N = non-first-line nurses*.

### Binary Logistic Regression Analyses of Front-Line Doctors/Front-Line Nurses' Depression and Insomnia During the COVID-19 Pandemic

Complete random analysis of variance was performed on the samples of first-line doctors, first-line nurses, non-first-line doctors, and non-first-line nurses, and the results showed that there were statistically significant differences in PHQ-9, GAD-7, and ISI scale scores among the four groups (*F* = 9.73, *P* < 0.001; *F* = 9.32, *P* < 0.001; *F* = 23.45, *P* < 0.001). A *post hoc* test found that, compared with non-front-line doctors, first-line doctors showed more severe depression (p=0.03) and insomnia (*p* = 0.001) problems; compared with non-first-line nurses, first-line nurses showed more severe depression, anxiety, and insomnia problems (*p* < 0.001). In addition, it is worth noting that front-line nurses suffer more from insomnia than front-line doctors. It can be seen that depression and insomnia are the two major problems for front-line medical staff. Age, education level, gender, smoking, drinking, and GAD-7 score (anxiety level) were used as independent variables, and PHQ-9 score (depression level) and ISI score (insomnia level) were used as dependent variables, respectively. We undertook regression analysis to explore possible influencing factors. The results are shown in Table 5 (depression) and Table 6 (insomnia). The age and anxiety level of the front-line doctors are the factors that affect the depression of front-line doctors. The education and anxiety level of the front-line doctors are the factors that affect the insomnia of front-line doctors.

**Table 5:** Influencing factors of depression in Doctor FL.

	**Beta**	**Wald**	***P***	**OR**	**95%**	
					**Inferior**	**Superior**
Age	−0.34	10.31	0.001	0.71	0.57	0.87
Education	0.18	2.80	0.09	1.20	0.97	1.49
Sex	0.34	2.77	0.09	1.41	0.94	2.11
Smoke	−0.20	0.69	0.41	0.82	0.51	1.31
Drink	−0.48	2.49	0.11	0.62	0.34	1.12
GAD-7(anxiety)	0.54	263.63	<0.001	1.72	1.61	1.84

*Doctor FL, front-line doctors; OR, odds ratio; CI, Confidence Interval*.

**Table 6:** Influencing factors of insomnia in Doctor FL.

	**Beta**	**Wald**	***P***	**OR**	**95%**	
					**Inferior**	**Superior**
Age	0.15	2.30	0.13	1.16	0.96	1.39
Education	0.24	5.84	0.02	1.27	1.05	1.55
Sex	−0.22	1.32	0.25	0.80	0.55	1.17
Smoke	−0.23	1.18	0.28	0.79	0.52	1.21
Drink	−0.10	0.13	0.72	0.90	0.52	1.57
GAD-7(anxiety)	0.39	224.23	<0.001	1.48	1.41	1.56

*Doctor FL, front-line doctors; OR, odds ratio; CI, Confidence Interval*.

We used age, education level, gender, smoking, drinking, and GAD-7 score (anxiety level) as independent variables, while the PHQ-9 score (depression level) and ISI score (insomnia level) were dependent variables respectively. Regression analysis was used to explore possible influencing factors. The results are shown in Table 7 (depression) and Table 8 (insomnia). The age and anxiety level of the front-line nurses are the factors that affect the depression of front-line nurses. The anxiety level of the front-line nurses will significantly affect their sleep status.

**Table 7:** Influencing factors of depression in Nurse FL.

	**Beta**	**Wald**	***P***	**OR**	**95%**	
					**Inferior**	**Superior**
Age	−0.32	10.86	0.001	0.72	0.60	0.88
Education	0.07	0.38	0.54	1.07	0.86	1.34
Sex	−0.34	0.52	0.47	0.71	0.28	1.79
Smoke	0.11	0.03	0.86	1.11	0.33	3.71
Drink	−0.07	0.01	0.91	0.93	0.28	3.11
GAD-7 (anxiety)	0.57	370.41	<0.001	1.77	1.67	1.87

*Nurse FL, front-line nurses; OR, odds ratio; CI, Confidence Interval*.

**Table 8:** Influencing factors of insomnia in Nurse FL.

	**Beta**	**Wald**	***P***	**OR**	**95%**	
					**Inferior**	**Superior**
Age	−0.09	1.16	0.28	0.91	0.78	1.08
Education	0.17	2.77	0.10	1.18	0.97	1.43
Sex	−0.52	1.57	0.21	0.60	0.26	1.34
Smoke	0.24	0.19	0.66	1.27	0.44	3.69
Drink	−0.99	3.32	0.07	0.37	0.13	1.08
GAD-7(anxiety)	0.39	316.05	<0.001	1.48	1.42	1.55

*Nurse FL, front-line nurses; OR, odds ratio; CI, Confidence Interval*.

## Discussion

Demographic information showed that the front-line medical staff as a group were younger and more educated. In this study, the levels of depression, anxiety, and insomnia of front-line medical staff were significantly higher than those of non-front-line, which was consistent with the previous research results (Spoorthy, 2020). This illustrates that front-line medical staff are under greater psychological pressures, possibly because of the more serious epidemic environment, larger workloads, and greater treatment pressures.

We further found that there were significant differences between doctors' and nurses' psychological statuses. Front-line nurses had the highest level of insomnia and the worst sleep status. It could be seen that, compared with front-line doctors, front-line nurses were under greater psychological pressure. One study ruled out the gender effect in making this difference (Kramer et al., 2020). A previous study on the sources of stress of nurses in the infectious disease department showed that stress mainly came from the following aspects: worry about making mistakes at work, lack of support from family and friends, the high demands on nurses from doctors, and fear of contracting the disease (Zhao, 2020). In daily medical care, nurses get in touch with patients more frequently than doctors. During the pandemic, the length of contact with patients and the level of exposure to the patient (mental) burden seem to be the crucial factors that cause nurses to have much heavier workloads and suffer greater mental pressures (Miriam et al., 2021).

The results of this study illustrate that depression and insomnia were the two main psychological problems faced by front-line medical staff. In order to find the influencing factors behind them, we undertook further exploration. Similar to previous findings, anxiety and insomnia had a high comorbidity rate and appeared in a bidirectional relationship (Huang et al., 2018). We found that anxiety was a predictor of depression and insomnia in medical staff, and higher anxiety levels were associated with more severe depression and insomnia. According to the diagnostic criteria of DSM-5 (the latest version of the American Psychiatric Association, APA), sleep disturbance is a symptom of generalized anxiety disorder, correspondingly, mood disorders, especially anxiety, are common causes of insomnia (Harvey, 2002). Neurological research has provided physiological evidence for this and neuroimaging showed that sleep deprivation reduces functional connectivity between the medical prefrontal cortex and amygdala, increasing the amygdala's response to negative stimuli, which may lead to anxiety (Yoo et al., 2007). Thus, it can be seen that the prefrontal limbic network plays an important role in the cognitive control of the emotions of human beings, controlling the response to negative emotions of the amygdala. Sleep deprivation may reduce control of the prefrontal limbic network to the amygdala so that low-level negative events also lead to greater negative emotional experiences. In addition, sleep deprivation increased cortisol production, which also led to anxiety (Wright et al., 2015). Cortisol concentration is positively correlated with cortisol stress response (Kidd et al., 2014). A certain concentration of cortisol can mobilize the body's energy to help individuals cope with stressful events (Jacobs et al., 2007). Therefore, when cortisol is always at a high level of concentration, the individual is always in a state of long-term stress preparation, resulting in long-term energy loss, which will adversely affect the individual's physical and mental health over time. A previous investigation found that medical staff who worked more than 8 h a day were more likely to suffer from symptoms of insomnia (Huang et al., 2021). Therefore, long-term sleep deprivation can lead to anxiety among medical staff. The COVID-19 pandemic has brought unprecedented pressures and challenges to medical staff (Greenberg et al., 2020). Although the National Health Commission of China issued a notice on the basic principles of emergency psychological crisis interventions for COVID-19, the Mental Health Care Services for medical staff remain unresolved (Xia et al., 2021). Front-line medical staff face multiple physical and mental pressures. The long-term heavy workload of front-line medical staff may lead to circadian rhythm disorders, meaning they are more likely to go through psychological problems.

This study also found that age was a protective factor for depression in front-line medical staff (OR = 0.71 <1, *P* = 0.001 <0.05, Beta = −0.34). In other words, when facing the same situation as their colleagues, people in the middle age group had lower depression levels than younger people, which was in line with previous studies reporting that older respondents were less susceptible to anxiety and depression disorders than younger people (Su et al., 2007; Huang and Zhao, 2020). Medical staff who are in the middle ages may have rich work experiences and have dealt with much more complex situations, meaning they are able to handle emergencies more calmly and can better adjust their psychological conditions than those who are younger during the epidemic (Su et al., 2021). Conversely, educational level was a risk factor for insomnia (OR = 1.27, *P* = 0.02 <0.05, Beta = 0.24). Medical staff who are well-educated have received more professional, comprehensive, and higher-level medical training, which helps them use advanced skills to process difficult problems. Compared with previous research, the results above were consistent in some respects (Wang et al., 2020); however, this study did not find a gender difference in the psychological problems experienced by medical staff. A possible reason for this is that this study focused on exploring differences in psychological status between front-line and non-front-line medical staff. Because of the similarity in work circumstances of front-line medical staff, meaning that gender difference was not so evident.

There were a few limitations in this study. Firstly, in this research, questionnaires were used for investigation, and participants presented their states through self-report, which would inevitably lead to research bias. Therefore, if regulation allows, future studies can explore the neural mechanism of mental stress during the epidemic in an experimental way to provide a reference for clinical practice. Secondly, although the paper took samples from several regions in China to make the sampling more comprehensive, due to the difficulty of obtaining relevant demographic information, there may be sampling equilibrium deviation, which can be considered to optimize in future research. Last but not least, this study conducted different tests on the scale scores of depression, anxiety, and insomnia among FL and non-FL medical staff, but did not further explore whether FL medical staff were more stressed than non-FL due to their jobs or demographics. Therefore, future research could further explore this issue by doing sensitivity analysis with the help of economic knowledge.

In a word, the COVID-19 outbreak has had a severe impact on people throughout society, not only taking a toll on their physical health but their mental health, especially on the medical staff who save patients day and night in hospitals and snatch lives from the hands of death. These findings can identify factors that influence the mental health of medical staff and the characteristics of vulnerable populations, which in turn could help the government and relevant authorities develop more targeted interventions. In addition, while calling on the government and health systems to provide psychological intervention measures, it is suggested to carry out daily mental health monitoring for medical staff to improve their working and life quality.

## Conclusion

To conclude, this study revealed that the severity of psychological problems of front-line medical staff was significantly higher than that of non-front-line medical staff. Depression and insomnia were the two main psychological symptoms that front-line medical staff were prone to, especially front-line nurses, with age, education, and anxiety level being influencing factors. We hope that this study will help provide quantitative evidence to establish further psychological interventions for medical staff, especially those on the front-line.

## Data Availability Statement

The original contributions presented in the study are included in the article/supplementary material, further inquiries can be directed to the corresponding authors.

## Ethics Statement

The studies involving human participants were reviewed and approved by Ethics Committee of Meizhou People's Hospital. The patients/participants provided their written informed consent to participate in this study. Written informed consent was obtained from the individual(s) for the publication of any potentially identifiable images or data included in this article.

## Author Contributions

DZ, HL, and LX are responsible for research conception, questionnaire issuance, data collection, and article writing. ZZ, JH, and XL assist to complete the research process. CL is responsible for the communication of the paper with HZ. All authors contributed to the article and approved the submitted version.

## Conflict of Interest

The authors declare that the research was conducted in the absence of any commercial or financial relationships that could be construed as a potential conflict of interest.

## Publisher's Note

All claims expressed in this article are solely those of the authors and do not necessarily represent those of their affiliated organizations, or those of the publisher, the editors and the reviewers. Any product that may be evaluated in this article, or claim that may be made by its manufacturer, is not guaranteed or endorsed by the publisher.
